# Role of Pediatric Dentistry in Healthcare and Economy: A Global Perspective

**DOI:** 10.12669/pjms.41.2.11710

**Published:** 2025-02

**Authors:** Amjad H Wyne

Early dental health care leads to better health outcomes in adulthood. Pediatric dentistry is an age-defined specialty that provides both primary and comprehensive preventive and therapeutic oral health care to infants and children through adolescence, including those with special health care needs.^1^ Oral health is a fundamental component of overall health, influencing physical development, well-being, and quality of life.^2^ Therefore, it is of utmost importance to realize that children’s dentistry must be a priority over other oral health care specialties in national health care systems.

A recent (26-29 November 2024) World Health Organization (WHO) global conference held in Bangkok issued a “Bangkok Declaration” that was titled “No Health Without Oral Health: Towards Universal Health Coverage for Oral Health by 2030”.^3^ The declaration acknowledged with great concern that oral diseases affect 3.5 billion people globally, which poses significant public health challenges for all WHO Member States and highlights the critical need to address oral diseases and conditions as part of the broader burden of non-communicable diseases.

An early routine dental check-up visits to pediatric dentist prevent oral health problems like dental caries, that is one of the most common childhood diseases.^4^ The WHO Global Oral Health Status Report (2022) estimated that oral diseases affect close to 3.5 billion people worldwide, with 3 out of 4 people affected living in middle-income countries. Globally, an estimated 514 million children suffer from caries of primary teeth.^4^ Regular check-up dental visits to a pediatric dentist allow prevention, early diagnosis and treatment of dental diseases, reducing the risk of complications in future.^1^

Pediatric dentists are able to educate children and their parents about optimal oral hygiene routine and healthy feeding/dietary habits resulting in lifetime of better oral health. These habits, when developed early, lead to fewer dental diseases in adulthood, thus reducing the strain on healthcare systems of a country. Children are in a critical stage of dental development, and pediatric dentists are trained to identify and address issues like malocclusions or abnormal tooth eruptions. Early intervention can prevent more severe problems and expensive corrective treatments in the future.

Good oral health in children can improve general health outcomes. Dental infections, if left untreated, can lead to complications like infections in other parts of the body, malnutrition due to difficulty in eating, or speech problems. Pediatric dentistry plays a key role in ensuring that oral health does not negatively affect a child’s overall development.

Poor oral health is linked to several health problems, including heart disease, diabetes, and respiratory infections.^5^ In children, untreated dental issues can lead to pain, difficulty in eating, and even stunted growth. Pediatric dentistry helps mitigate these risks by addressing problems early.^5^

By emphasizing preventive care, pediatric dentistry reduces the likelihood of severe dental problems that require costly interventions. This not only decreases healthcare spending but also improves productivity, as children with better oral health are more likely to attend school and perform well academically.

The total estimated global economic impact of oral conditions was US $710B in 2019, of which US $387B was due to direct costs and US $323B was due to productivity losses for the five main oral conditions.^6^ Low-income countries spent an average of US $0.52 per capita on dental care, while high-income countries spent an average of US $260 per capita, a 500-fold difference. 6 These findings suggest that oral conditions continue to be an enormous economic burden to individuals and societies.^6^

There is a global need of provision of oral health care to children. At National level, according to the 2023 Census Report, 47% of Pakistan’s population is under the age of 18 years. This makes Pakistan one of the world's countries with the youngest populations due to its high fertility rate. Considering the lack of trained pediatric dentists and minimal undergraduate training in pediatric dentistry, urgent efforts have to be made to ensure appropriate training in pediatric dentistry both at undergraduate and postgraduate levels to ensure provision of quality oral health care to its young population. Oral health education institutions need to address the dearth of trained pediatric dentists in the country by taking initiatives in starting postgraduate level programs in pediatric dentistry This will assist in controlling unmet needs of oral health care in pediatric population including those with special care needs.

Pediatric dentistry is critical for ensuring the oral and overall health of future generations. It helps prevent dental diseases, promotes healthy habits, and addresses developmental concerns early, leading to better health outcomes. By prioritizing pediatric dental care, countries (especially developing countries) can reduce healthcare costs and contribute to the well-being of their population, ultimately fostering healthier communities and economies.
